# Fatal H1N1-Related Acute Necrotizing Encephalopathy in an Adult

**DOI:** 10.1155/2011/562516

**Published:** 2011-07-05

**Authors:** Yu-Jin Lee, D. Scott Smith, Vivek A. Rao, Robert D. Siegel, Jon Kosek, Carol A. Glaser, Alexander C. Flint

**Affiliations:** ^1^Department of Human Biology, Stanford University, Stanford, CA 94305, USA; ^2^Department of Infectious Disease and Geographic Medicine, Kaiser Permanente, Redwood City, CA 94063, USA; ^3^Department of Neuroscience, Kaiser Permanente, Redwood City, CA 94063, USA; ^4^Department of Microbiology and Immunology, Stanford University, Stanford, CA 94305, USA; ^5^Department of Pathology, Veterans Affairs Palo Alto Health Care System, Palo Alto, CA 94304, USA; ^6^Center for Infectious Diseases, California Department of Public Health, Richmond, CA 94804, USA

## Abstract

Acute necrotizing encephalopathy (ANE) is a severe neurological complication of influenza infection, including H1N1 influenza. Many cases of ANE have been reported in the pediatric literature, but very few cases have been described in adults. The cause of ANE remains unknown—the influenza virus is not known to be neurotropic, and evidence of direct viral involvement of the central nervous system (CNS) has not been demonstrated in the limited cases of ANE in which pathological specimens have been obtained. Here we report a fatal case of ANE from H1N1 influenza infection in an adult. Neuroimaging and postmortem analysis both showed widespread brain edema, necrosis, and hemorrhage, but molecular studies and postmortem pathology revealed no evidence of direct viral involvement of the CNS. This case of fatal ANE in an adult is consistent with the hypothesis generated from pediatric cases that the host immune response, and not direct viral invasion of the CNS, is responsible for pathogenesis of ANE.

## 1. Introduction

Acute necrotizing encephalopathy (ANE) is a rare central nervous system (CNS) complication of influenza that is characterized by altered mental status and seizures, and often leads to profound disability or death [1]. ANE associated with influenza A was originally reported in pediatric patients of Asian descent [1], but subsequent reports worldwide indicate that ANE is not limited to a particular race/ethnicity [2–4] and can occur, albeit rarely, in adults [5–8]. During the 2009 influenza A (H1N1) pandemic, many pediatric ANE cases were reported but adult cases were uncommon [7, 9]. In this report, we present the first case of H1N1 encephalopathy progressing to brain death in an adult patient. The radiographic and pathologic findings in this fatal adult case of ANE are consistent with reports from pediatric cases. Molecular analyses of cerebrospinal fluid (CSF) and brain tissue from this case support the hypothesis that ANE occurs as a result of secondary immune-related mechanisms rather than direct invasion of the CNS by the influenza virus.

## 2. Clinical Case

A 27-year-old woman with a history of obesity and asthma was admitted to hospital with a diagnosis of H1N1 influenza A pneumonia and acute respiratory distress syndrome (ARDS). After a prodrome of nonproductive cough and chest tightness, she presented to the emergency department with worsened shortness of breath and subsequently required intubation and mechanical ventilation for hypoxic respiratory failure. On the day of admission, a nasopharyngeal sample was positive for influenza A by reverse transcriptase-polymerase chain reaction (RT-PCR). She had not received seasonal influenza or 2009 H1N1 pandemic flu vaccinations prior to her illness.

Antiviral therapy with oseltamivir was administered for a 10-day initial course. Given a persistently positive RT-PCR for influenza A in 3 samples over 3 weeks and worsening of her ARDS despite the course of oseltamivir, she subsequently received the investigational antiviral agent peramivir intravenously after consent was obtained from her family. The patient's severe ARDS required treatment with a course of intravenous methylprednisolone and neuromuscular paralysis to facilitate appropriate mechanical ventilation. On day 11 of her hospitalization, the patient developed acute oliguric renal failure and required hemodialysis for several weeks. Tracheostomy was performed approximately one month into her hospitalization. 

Five weeks into her hospitalization, as her sedation was lightened in the context of improved oxygen requirements, the patient was found to have altered mental status and quadriparesis. The next day, she became unarousable with nonreactive pupils, absent corneal reflexes, and absent motor response to noxious stimuli. An unenhanced head computed tomography (CT) showed diffuse cerebral white matter edema, with posterior fossa edema causing effacement of the fourth ventricle and obstructive hydrocephalus. The CT also showed a small intraparenchymal hemorrhage in the left occipital lobe. The patient was urgently transferred to the Neuroscience Intensive Care Unit at our Neurosurgical Center, where a ventriculostomy was placed.

**Figure 1:**
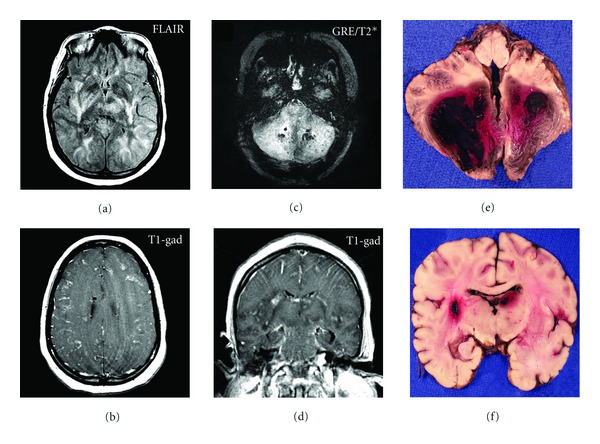
(a) FLAIR MRI sequence demonstrates widespread abnormal signal in the subcortical white matter, internal capsule, putamen, globus pallidus, and thalamus bilaterally. (b) T1 post-gadolinium axial MRI sequence demonstrates diffuse leptomeningeal enhancement over the convexities bilaterally and foci of parenchymal enhancement within the cortex bilaterally. (c) Gradient echo (GRE/T2*) MRI sequence demonstrates hemorrhage in the deep cerebellar white matter bilaterally (mottled black signal, representing magnetic susceptibility). (d) T1 post-gadolinium coronal MRI sequence demonstrates basal leptomeningeal enhancement surrounding the brainstem. (e) Postmortem axial section through the brainstem and cerebellum demonstrates severe bilateral hemorrhage and necrosis of the cerebellar hemispheres. (f) Postmortem coronal section demonstrates widespread but patchy areas of hemorrhage and necrosis in the deep gray nuclei bilaterally and the cerebral hemispheres bilaterally.

Magnetic resonance imaging (MRI) of the brain illustrated diffuse abnormal T2/FLAIR hyperintensities with swelling in the gray matter and subcortical white matter throughout the bilateral cerebral hemispheres, cerebellum, brainstem, and thalami (Figures 1(a)–1(d)). There was also evidence of edema in the bilateral lentiform nuclei and posterior limb of the internal capsules. There were signs of tonsillar herniation and upward cerebellar herniation. Diffuse leptomeningeal enhancement and patchy parenchymal enhancement were present throughout the bilateral cerebellar hemispheres, right basal ganglia, and the left ventromedial thalamus. Multiple areas of microhemorrhage were seen on gradient echo sequences at the gray-white junction in the cerebrum and cerebellum. The CT and MRI findings were consistent with a diagnosis of ANE [1].

Cerebrospinal fluid (CSF) obtained from the patient's ventriculostomy showed 7 white blood cells per microliter (97% polymorphonuclear leukocytes, 3% lymphocytes), protein level of 78 mg per deciliter, and glucose level of 65 mg per deciliter. Molecular analyses of the CSF and serum samples were negative for the virus. 

After treatment of the patient's obstructive hydrocephalus by ventriculostomy, no clinical improvement was observed. Given the possibility of an inflammatory or parainfectious etiology for the patient's acute necrotizing meningoencephalitis, treatment with intravenous immunoglobulin was begun and steroid therapy was restarted. Despite these treatments, her neurological examination continued to deteriorate and the patient progressed to brain death. 

Postmortem examination revealed evidence of severe influenza pneumonia and advanced interstitial pneumonitis with fibrosis. The brain showed extensive and widespread edema, necrosis, and hemorrhage (Figures 1(e)–1(f)). Multiple brain regions demonstrated diffuse edema, venous congestion, and multifocal areas of hypoxic ischemic neuronal injury. The internal capsule and basis pontis demonstrated intravascular fibrin thrombi surrounded by cellular debris with early necrosis. Beta-amyloid precursor (beta-APP) immunostains in these regions demonstrated widespread axonal injury associated with the vascular lesions. The thalamus showed venous congestion and parenchymal hemorrhage without associated inflammation. The corpus callosum had small necrotic foci with a macrophage response and also showed evidence of marked axonal injury on the beta-APP immunostains. The cerebellar hemispheres were necrotic with associated hemorrhage (Figure 1(e)), and the basilar leptomeninges were congested.

Molecular analyses of multiple samples of postmortem brain tissue were negative for the virus.

## 3. Discussion

We describe an adult patient with fatal acute necrotizing encephalopathy (ANE) associated with 2009 H1N1 influenza infection. The findings on neuroimaging and postmortem examination in this case support the concept that ANE is a clinically recognizable form of severe necrotizing encephalopathy that has similar characteristics in adults and children. Molecular analysis of CSF and brain tissue support the hypothesis that ANE is not caused by direct invasion of brain by the influenza virus, but instead may represent a consequence of the immune response to the virus [10].

In addition to respiratory illness, influenza virus infections can be associated with a number of different neurological complications [7]. Among the neurological syndromes reported, the most devastating is ANE, which presents as a rapid neurological decline following influenza infection [10]. ANE is associated with high morbidity and mortality [7]. The term ANE was first coined by Mizuguchi following reports of encephalopathy associated with influenza infection in East Asia [1]. Initially described as a neurological syndrome in pediatric patients of Asian descent, influenza-associated ANE is now recognized worldwide^39^. Although ANE appears to have a predilection for the pediatric age group, a small number of cases in adults have also been reported [5–7]. The characteristic neuroimaging findings of ANE are bilateral, multifocal lesions in the thalami, cerebral and cerebellar deep white matter, brainstem tegmentum, and putamen [1, 10]. Common clinical signs and symptoms include altered mental status, seizures, ophthalmoparesis, quadriparesis, and delayed parkinsonism among survivors. During the 2009 H1N1 pandemic, a wide range of neurological complications were reported in association with influenza infection, including ANE in several pediatric cases and in some adults [3, 4, 7, 8, 11–14]. 

Neuroimaging in ANE characteristically shows abnormalities in the subcortical and cerebellar white matter, with microhemorrhages and abnormal enhancement [1, 10]. Our patient's MRI showed edema throughout the bilateral cerebral hemispheres, cerebellum, brainstem, and thalami. The gradient echo (GRE) sequence showed microhemorrhages at the gray-white junction in the cerebrum and cerebellum. Postmortem brain sections illustrated hemorrhage and necrosis in both the cerebral and cerebellar hemispheres. These findings were consistent with a diagnosis of ANE and support the hypothesis that characteristic neuroimaging findings in ANE are seen in both pediatric and adult cases [1]. 

The influenza virus has not been detected in the CNS in ANE cases analyzed to date, including in CSF and brain tissue samples from pediatric cases [2–4, 11, 15] and only in CSF samples from adult cases [5, 7]. In our patient, RT-PCR for influenza virus was negative on both CSF and brain tissue, and postmortem analysis showed no evidence for viral invasion of the CNS such as cellular inclusions. Our postmortem pathological analysis and molecular biological analysis results thus provide further evidence that direct viral invasion of the CNS may not be involved in the pathogenesis of ANE [2, 11]. The lack of viral detection in CSF and brain tissue in this adult patient further supports the notion that ANE is not a fundamentally different condition in pediatric and adult patients. However, it should be noted that the RT-PCR may have been negative as a result of the interval between the initial influenza infection and the time CSF and brain samples were obtained. It is difficult to know at what point this patient developed the neurological complications, but it is likely that it was relatively early in her hospital course. ANE usually occurs within a few days of the onset of respiratory illness [1, 5, 9]. Because she required neuromuscular paralysis for ventilatory management of her ARDS, daily sedation interruptions to facilitate neurologic evaluation were not possible. 

This case typifies the rare but devastating complication of ANE associated with influenza (H1N1) infection. In the wake of the recent 2009 H1N1 pandemic, awareness of this rare but significant complication is important. In order for early detection of ANE, closer neurological monitoring of patients with ARDS is necessary. Given the mounting evidence that this dramatic syndrome is not caused by direct viral invasion of the CNS, better understanding of the pathophysiology of this severe inflammatory condition is needed so that appropriate prevention or treatment measures may be applied.

##  Funding

The authors declare that there are no sources of funding to disclose.

##  Conflict of Interests

The authors declare no conflict of interests.
